# A Literature Review on Biofilm Formation on Silicone and Poymethyl Methacrylate Used for Maxillofacial Prostheses

**DOI:** 10.7759/cureus.20029

**Published:** 2021-11-30

**Authors:** Ashok Kumar, Madhan Kumar Seenivasan, Athiban Inbarajan

**Affiliations:** 1 Prosthodontics and Crown & Bridge, Sri Ramachandra Dental College, Chennai, IND; 2 Prosthodontics, Sri Ramachandra Institute of Higher Education and Research, Chennai, IND

**Keywords:** silicone elastomers, commensal, microorganisms, biofilm, prosthesis

## Abstract

Silicone elastomers are considered the most suitable maxillofacial materials for extraoral prostheses to date due to their superior physicochemical properties. The aim of this review was to describe the characteristics of biofilm formation on silicone and polymethyl methacrylate used for maxillofacial prostheses and review different strategies of biofilm management for silicone maxillofacial prosthesis. A structured literature search was conducted using the following databases - PubMed, Google Scholar, ScienceDirect, LILACS, IndeMED, OVID, EMBASE, NIH Clinical Trials - for reports related to the biofilms. English language articles were only included in the study. Biofilms induced various systemic infections if they are not treated at an early stage. Biofilms are formed due to various reasons like fungal, bacterial and mixed infections of the patient and also due to prosthetic appliances. The manual or mechanical pressure physically removes the biofilm and most biofilm molecules from surfaces. Treatment must be given with utmost caution and concern irrespective of the presence or absence of biofilm. With regards to the materials used for fabricating maxillofacial substitutes, it has been defined that both acrylic resin and silicone may harbour microorganisms, however, the larger porosities in silicone make it vulnerable to microbial adhesion. The major limitations of these materials are that they have numerous porosities on their surface and, along with the modification of the anatomy of the facial tissues as a result of the lesion, may compromise the natural balance of the microbial flora, favouring microbial colonization and formation of biofilms.

## Introduction and background

The area of maxillofacial prosthodontics has transformed the way of patients with oral and maxillofacial cancer and trauma are cared for and provide a good quality of life after surgery. The use of a maxillofacial prosthesis is intended to help patients who have had major facial mutilation regain function and appearance. Patients' quality of life improves significantly as a result of the prosthesis assisting them in performing day to day functions like chewing and speaking. The prosthesis is fabricated with inexpensive materials, and its usage is simple and non-invasive [1]. Silicone, polymethyl methacrylate (PMMA), and titanium are the most often utilised materials for obturator prostheses [2]. When reconstructive surgery is not possible, face prostheses are made to cover defects in disfigured individuals due to acquired or congenital problems in the facial area. By using intrinsic and extrinsic stains, silicone elastomer prosthetics can be fabricated to match the colour of the skin. Adhesives or percutaneous implants, in combination with a bar/clip or magnet, are commonly utilised for retention [3]. The existence of microbial colonies on the surface of the prosthesis is a major drawback. This colonisation may be exacerbated as a result of the lesion's alteration of the morphology of the facial tissues, jeopardising the microbial flora's natural balance [3]. Microorganisms can cling to the surface and then infiltrate and survive in the interior of the prosthesis due to the presence of pores, fissures, and structural defects created by the discharge of gases during the polymerization process. Due to the presence of pores, fissures, and structural defects generated by gas discharge during the polymerization process, microorganisms can cling to the surface and then infiltrate and survive in the interior of the prosthesis. Local or systemic infections can develop from microbial exposure; however, the severity of the infection is determined by the patient's overall health and the pathogenicity of the bacteria [4]. The requirement of getting radiotherapy in these patients increases the risk of opportunistic infections. The establishment of proper hygiene requires knowledge of the biofilm that forms on maxillofacial prostheses and their closely related tissues.

## Review

Materials and methods

**Figure 1:**
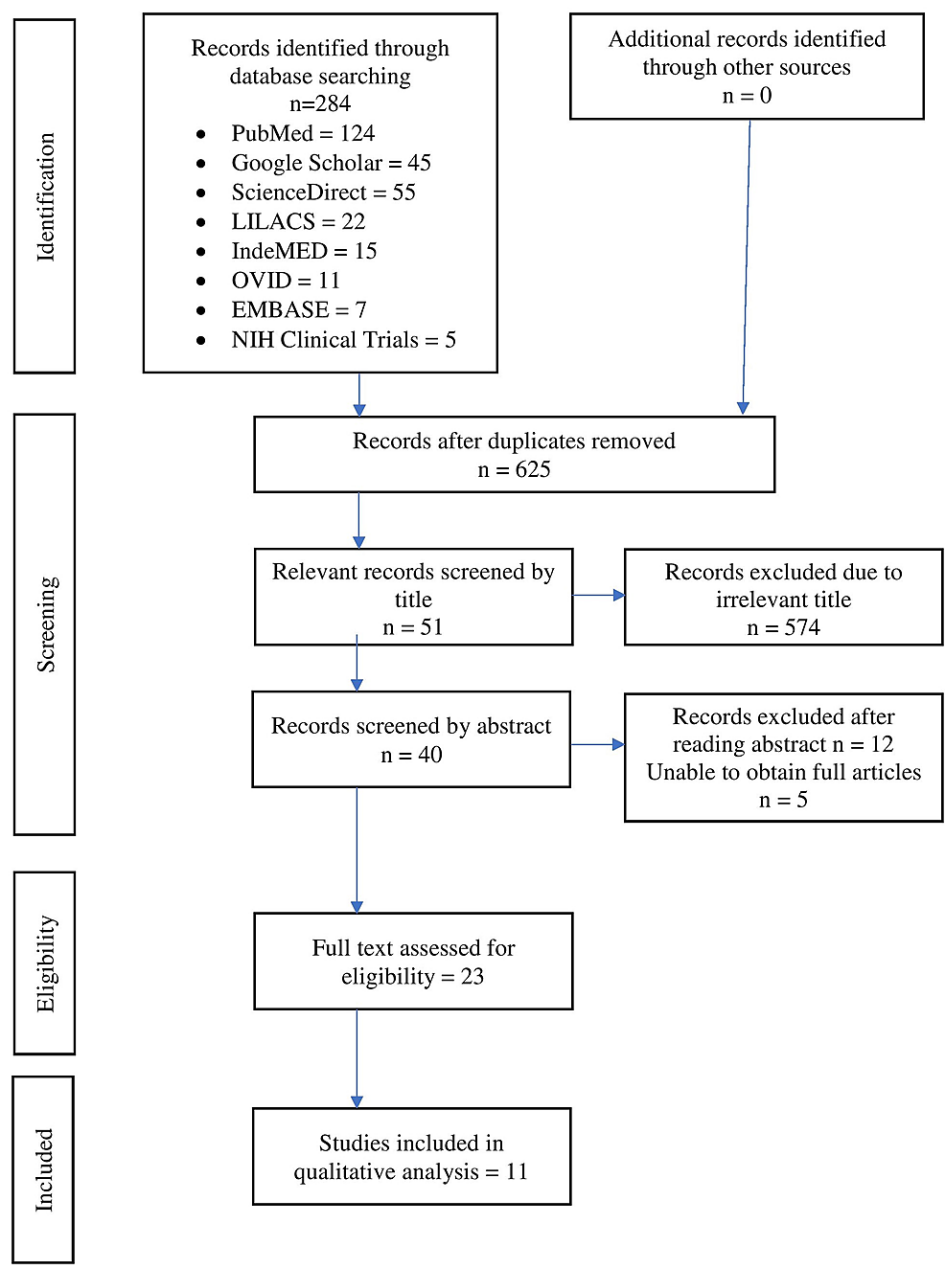
PRISMA flow chart PRISMA: Preferred Reporting Items for Systematic Reviews and Meta-Analyses

Literature published between the years 2000 to 2019 was searched for by three independent researchers from the following databases: PubMed, Google Scholar, ScienceDirect, LILACS, IndeMED, OVID, EMBASE, NIH Clinical Trials for reports related to the biofilms. The types of studies included were in vivo, in-vitro and narrative reviews with regards to normal oral microflora, microbiome, biofilms, and microflora of maxillofacial prosthesis. Case reports, animal studies, and those published in languages other than English were excluded from the study. The following keywords were used to develop the search strategy ((biofilms AND bacteria AND fungus AND maxillary defects AND polymers AND opportunistic infections) AND (PMMA OR silicones OR mixed biofilm OR obturator)) AND (microbiome OR microbial contamination) AND (immunocompromised OR immunocompromised patients) AND (soft liners OR resilient soft liners). The electronic database search yielded 1084 articles, and after excluding the duplicates, 625 records remained. Articles were removed after screening the titles that assumed that the title was descriptive of the study; 23 were retained following this full-text screening, resulting in the elimination of 12 papers. This is represented as a PRISMA (Preferred Reporting Items for Systematic Reviews and Meta-Analyses) chart in Figure 1. The inclusion criteria were applied to assess the eligibility of the record obtained. Eventually, 11 studies were included in this review and submitted to data extraction.

Biofilm and microbial adhesion

Biofilms that have collected on a solid surface are made up of bacteria and extracellular components. When bacteria cling to a surface, they release mostly insoluble gelatinous exopolymers, forming a three-dimensional matrix known as a biofilm [5]. Both commensal and pathogenic microorganisms produce biofilm-like aggregates that have been related to a variety of ailments. These disorders are usually caused by the presence of endothelium or epithelial lining that is entrenched on the surface of the skin or a prosthesis. Since germs forming as biofilms are less sensitive to topical treatments, antibiotics, and host defences than planktonic forms of the same microorganisms, biofilm formation and persistence have implications for the patient. Many biofilm infections are difficult to treat and manifest as chronic or recurrent illnesses. Biofilm infections can cause diseases such as uncultivable species, extended inflammation, delayed wound healing, rapidly acquired antibiotic resistance and the development of infectious emboli.

Stages of biofilm formation

Biofilm formation is the result of a combination of physical, biological, and chemical processes, each of which contributes differently depending on the current environmental and hydrodynamic circumstances. A bacterium's ability to cling to a surface and form surface-attached microbial communities, or biofilms, is divided into many phases [5]. The steps of biofilm formation are 1) attachment of the microorganism to a surface 2) colony development and extracellular polysaccharide secretion in conjunction with matrix assembly, and 3) dissemination of daughter biofilm cells. Microbial adherence to non-shedding surfaces occurs on substrata in the oral cavity and on the prosthesis in the initial stage. Cell-cell binding in microorganisms is thought to have a role in the integration of primary and secondary colonisers into oral biofilms and the formation of a network of interacting microbial cells [6]. The second phase of biofilm growth comprises the expansion of bacteria on the surface and the simultaneous production of an extracellular polymeric matrix [7]. The matrix binds the microbial cells into a mass and secures them to the underlying surface. This matrix acts as a "scaffold" for the biofilm colony and causes biofilm-mediated antimicrobial resistance by acting as a diffusion barrier or directly binding antimicrobial chemicals and blocking their access to the biofilm cells. The final stage of biofilm growth is cell separation from the biofilm colony and dissemination into the environment [8]. Biological dissemination, bacterial survival, and disease transmission are all critical during this stage of the biofilm life cycle [4].

Bacterial and fungal populations in biofilms

Bacterial Biofilms

Bacteria that normally occur as commensals aggregate and transform into biofilms due to the niche created by the presence of prosthesis, which provides a scaffolding platform for aggregation and can potentially cause serious diseases, particularly in the elderly or immunocompromised and immunosuppressed patients. Coagulase-negative bacteria like *Staphylococcus*, *Enterobacter cloacae, Pseudomonas aeruginosa, Pseudomonas aeruginosa, Staphylococcus aureus, *Coagulase-negative* Staphylococcus, Aggregatibacter actinomycetemcomitans, Streptococcus mutans, Staphylococcus epidermidis, Enterococcus faecalis *and* Escherichia coli* are the most common germs recovered from prostheses [2]. *Corynebacterium amycolatum, Propionibacterium avidum, Serratia marcescens, Proteus mirabilis, Klebsiella spp, *and *Proteus spp* are among the unusual species discovered in modest concentrations. On the surfaces of skin and prosthesis, *Stapylococcus aureus* has been discovered to be colonising. It's also a pathogenic bacterium that's been employed as an antibiotic efficacy indicator [2]. *Streptococcus mutans *is a gram-positive bacterium that is important in the early stages of biofilm colonization in dental caries. *Enterococcus faecalis* is a gram-positive bacterium linked to apical periodontitis, recurrent endodontic infections, and invasive illnesses in the oral cavity. Despite being a common resident of the digestive tract, *Escherichia coli*, a gram-negative bacterium, is considered a transitory bacterium in the oral cavity that is responsible for the initial adhesion of yeast on various surfaces and can cause mild to severe infections depending on the immune system's situation. Furthermore, gram-negative systemic infections are dangerous because they can produce endotoxins and toxic cytokines [6]. Antimicrobial resistance, in combination with the rising prevalence of multi-resistant bacteria, particularly methicillin-resistant *Staphylococcus aureus*, can make systemic microbial infection chemotherapy even more difficult. *Pseudomonas aeruginosa* is a bacterium that can be present in the environment and is frequently linked to pneumonia in hospitalised or institutionalised patients [5].

Fungal Biofilms

Infections caused by fungi in biofilms are the most common type of infection, especially among patients who require a prosthesis and are immunocompromised. *Candida albicans* is a common commensal found in 30 to 70 percent of people who appear to be healthy [9]. *Candida glabrata, Candida tropicalis *and *Candida parapsilosis* are other *Candida *species that typically infect prostheses [10]. *Candida *strains may operate as an opportunistic pathogen in immunocompetent and immunosuppressed persons under predisposing conditions, causing severe and recurring mucosal infections as well as lethal invasion infections [11]. *Candida *biofilms arise when the fungus clings to a surface, such as a denture or other sort of prosthesis, and then evolves into a complex three-dimensional structure made up of yeast, germ tubes, and juvenile hyphae, all wrapped in an extracellular polysaccharide matrix [12]. When scanning electron microscopy is used to analyse *Candida albicans* biofilms, it reveals a variety of morphological shapes [13]. In vitro, yeast cells adhere to each other first, followed by germ-tube development after 3 to 6 hours. After incubation for up to 48 hours, fully complete biofilms consist of a dense network of yeasts, hyphae, and pseudohyphae, albeit the presence of hyphal forms is dependent on parameters such as the growth media and substratum [13]. Adhesion is often the first stage of infection for members of the genus *Candida*, and there are a variety of interactions between these fungi and other species. *Candida dubliniensis* has been found to interact with the oral bacterium *Fusobacterium nucleatum*, whilst *Candida albicans* and *Candida tropicalis* have been found to interact with *Streptococcus gordonii*. When compared to planktonic *Candida *cells, this relationship may allow them to survive in mixed oral habitats and boost resistance to antifungal medications.

Mixed Biofilms

*Staphylococci,* particularly *Staphylococcus epidermidis* and *Staphylococcus aureus*, are responsible for the majority of prosthesis-related bacterial infections. Maxillofacial implant infections caused by fungi are less prevalent, but aggressive [13]. Pathogenic *Candida *species, particularly *Candida albicans*, are the most common cause of these illnesses. In these mixed biofilm communities of species, antibacterials and antifungals are difficult to treat. Mixed biofilms may be more drug-resistant due to their more intricate matrix structure. Antibiotic resistance profiles have also been suggested to alter in mixed infections [6]. Microbial interactions in mixed-species habitats, on the other hand, appear to behave differently from those in monospecies ecosystems [14]. There are a variety of processes by which fungi and bacteria interact with each other. Bacterial effects on fungal development and survival are influenced by a variety of factors, including bacterial and fungal species, the surrounding microbial population, and the host environment [15]. *Surface polysaccharides* have been discovered to have a crucial role in *Candida albicans *colonisation of bacterial biofilms and bacteria colonisation of *Candida albicans* biofilms. Mixed biofilms alter the physical and nutritional environment, as well as the amounts of oxygen and iron, which can alter how different species interact in a biofilm.

## Conclusions

It's crucial to conduct research that explains how microbes interact with surfaces. This is particularly critical for patients who have been immunocompromised as a result of cancer resection surgery and radiotherapy, as pathogenic biofilms are more likely to affect them. Hence, adequate knowledge of mechanisms of microbial adherence is critical for reducing the risk of local and systemic infections in patients undergoing cancer resection surgery. Biofilm formation is the result of a combination of physical, biological, and chemical processes, each of which contributes differently depending on the current environmental and hydrodynamic circumstances. Biofilms are continuously being researched. This is because biofilms are responsible for a significant number of deaths as a result of nosocomial infections associated with medical devices, as well as their complexity. Antimicrobial treatment resistance is also influenced by biofilms. Biofilms have been related to a reduction in the lifespan of various prosthetic materials, as well as the onset of oral illnesses such as denture stomatitis, material surface deterioration, and colour loss in the case of facial silicone prosthesis. More research is needed on colonisation, microbial adhesion to prostheses, and the materials used to make these prostheses.
